# Two coagulase-negative staphylococci emerging as potential zoonotic pathogens: wolves in sheep's clothing?

**DOI:** 10.3389/fmicb.2013.00123

**Published:** 2013-05-15

**Authors:** Meghan F. Davis, Christine L. Cain, Amy M. Brazil, Shelley C. Rankin

**Affiliations:** ^1^Environmental Health Sciences, Johns Hopkins Bloomberg School of Public HealthBaltimore, MD, USA; ^2^Dermatology and Allergy, University of Pennsylvania School of Veterinary MedicinePhiladelphia, PA, USA; ^3^Master of Public Health Program, Johns Hopkins Bloomberg School of Public HealthBaltimore, MD, USA; ^4^Clinical Microbiology, University of Pennsylvania School of Veterinary MedicinePhiladelphia, PA, USA

First described together in 1988, *S. lugdunensis* and *S. schleiferi* are coagulase-negative *Staphylococcus* (CNS) species that recently have emerged as potential zoonotic pathogens (Freney et al., 1988). *S. lugdunensis* typically has been associated with human disease, primarily skin infections and endocarditis, but recently also has been described as an animal pathogen (Frank et al., 2008; Rook et al., 2012). *S. schleiferi*, which may be coagulase negative (CNS: subsp. *schleiferi*) or coagulase positive (CPS: subsp. *coagulans*), typically has been associated with skin infections in dogs and cats, but recently has been described as a human pathogen (Kumar et al., 2007; Tzamalis et al., 2013). In this sense, we apply Calvin Schwabe's definition of *zoonosis* as “shared infection” of animals and man, without ascribing direction of transmission from one to the other (Schwabe, 1984).

While the ubiquitous nature of the genus *Staphylococcus* generally, and CNS specifically, enhances opportunities for infection, the genetic characteristics of *S. lugdunensis* and *S. schleiferi* make them worthy of closer scrutiny. Both of these pathogens historically have been considered opportunistic and susceptible to many antimicrobials, unlike methicillin-resistant *S. aureus* (MRSA) and the veterinary pathogen methicillin-resistant *S. pseudintermedius* (MRSP), for which multidrug resistance is an ongoing clinical concern. Both *S. schleiferi* and *S. lugdunensis* have been demonstrated to carry SCC*mec* elements, and *S. schleiferi* isolates have been identified recently as multidrug resistant (Roberts et al., 2005; Starlander et al., 2011; Cain, 2013; Penna et al., 2013). *S. lugdunensis* and *S. schleiferi* are examples of CNS often overlooked by routine clinical diagnostic protocols that may be emerging as drug-resistant pathogens.

*S. schleiferi* is divided into two subspecies, subsp. *schleiferi* (coagulase-negative) and subsp. *coagulans* (coagulase-positive), which behave similarly in animals (Cain, 2013). They typically cause otitis or pyoderma in dogs, and rarely may be isolated from cats or birds (Abraham et al., 2007; Briscoe et al., 2009; Cain, 2013). Prevalence from skin, nares, mouth, or perineal carriage in the absence of disease is low, typically ≤2%, but is higher among diseased pets (Abraham et al., 2007; Griffeth et al., 2008; Beck et al., 2012; May et al., 2012). Of concern is the propensity for clinical isolates to be methicillin resistant, with many veterinary studies in the last decade reporting rates of 50% or higher (Kania et al., 2004; Bemis et al., 2006; Vanni et al., 2009; Cain et al., 2011a,b; Penna et al., 2013). SCC*mec* IV has been described in clinical isolates of *S. schleiferi* subsp. *coagulans* from pets (Roberts et al., 2005). Among methicillin-resistant isolates, decreased susceptibility to erythromycin or fluoroquinolones has been observed (Intorre et al., 2007; Vanni et al., 2009; Cain et al., 2011b). One of these studies linked fluoroquinolone resistance to alterations in the *gyrA* gene (Intorre et al., 2007). Our experience at the Veterinary Hospital at the University of Pennsylvania suggests that a trend of increasing resistance may be occurring. In 2012, 14% of all *S. schleiferi* isolates from our microbiology laboratory were resistant to erythromycin, an increase from 9% in 2005; 16% of isolates were resistant to tetracycline, an increase from 6% in 2005; and notably, 14% of isolates were resistant to trimethoprim-sulfamethoxazole, while no resistance was observed in 2005 (Rankin unpublished data). These data suggest that increasing antimicrobial resistance is limiting treatment options for clinical *S. schleiferi* infections in animals.

Little attention has been paid to *S. schleiferi* in people outside of reports of outbreaks or individual cases (Freney et al., 1988; Vandenesch et al., 1994; Kluytmans et al., 1998; Hernandez et al., 2001; Kumar et al., 2007; Tzamalis et al., 2013). Although some reports have focused on surgical site infections, including those associated with pacemakers, others demonstrate a wider variety of sources, including wound or eye infections and meningitis (Hernandez et al., 2001; Tzamalis et al., 2013). Asymptomatic nasal or skin carriage in people with veterinary occupational contact also has been demonstrated, but prevalence may be ≤2% (Ishihara et al., 2010; Morris et al., 2010).

A paucity of data regarding genetic characterization of *S. schleiferi* limits discussion of its virulence. Genetically, CNS and CPS subspecies appear similar by pulsed-field gel electrophoresis (PFGE), particularly within methicillin-resistant strains (Yamashita et al., 2005; Cain et al., 2011a). Human outbreak isolates also have been found to be concordant by PFGE (Kluytmans et al., 1998). While these data suggest that the species may have low diversity, an insufficient number of strains have been typed globally to be conclusive. At the time of writing, the genome has yet to be sequenced. Like *S. aureus*, *S. schleiferi* may produce beta-hemolysin and exoenzymes, such as lipase, potentially associated with virulence (Hebert, 1990; Lambe et al., 1990; Linehan et al., 1992; Yamashita et al., 2005). It also shows adherence to glass, which may be relevant as a marker of propensity for infection of indwelling devices (Hebert, 1990). A murine model demonstrated that both *S. schleiferi* and *S. lugdunensis* formed abscesses at rates of 75–100%, similar to *S. epidermidis* but more frequent than *S. warneri* or *S. hominis* (all of which are CNS) (Lambe et al., 1990).

In contrast to *S. schleiferi*, *S. lugdunensis* primarily has been described as a human pathogen. Inguinal skin carriage is typical in people, as are skin infections in the groin, pelvic girdle or perineum (van der Mee-Marquet et al., 2003; Frank et al., 2008). Cardiovascular infections are common, with a 40% case-fatality rate among those reported in the literature, and *S. lugdunensis* also has been described in infections of the bone, joint, central nervous system, eye, and other sites (Frank et al., 2008).

Unlike *S. schleiferi*, *S. lugdunensis* tends to exhibit broad susceptibility to antimicrobial drugs, but may produce beta-lactamases (Hellbacher et al., 2006; Frank et al., 2008). One study of five clinical isolates in China noted that 80% of them produced beta-lactamase that all beta-lactamase-producing isolates demonstrated additional antimicrobial resistance phenotypes, and that 60% of isolates carried the *ermC* gene and were resistant to erythromycin (Liu et al., 2012). A few reports describe the occurrence of *mecA* in *S. lugdunensis*, including one report identifying the SCC*mec* type as V, similar to some community-associated *S. aureus* (Frank et al., 2008; Pereira et al., 2011; Liu et al., 2012). In addition, a novel penicillin binding protein mutation in a *mecA*-negative, beta-lactamase-resistant isolate has been described (Kotsakis et al., 2011). Laboratory discrepancies between presence of *mecA* and oxacillin disc testing may limit the reliability of disc methods (Ferreira et al., 2003). Development of resistance following antimicrobial treatment was described in a case report, but the frequency of acquisition of mobile genetic elements that confer resistance is unknown for *S. lugdunensis* (Kragsbjerg et al., 2000). Of note, many *S. lugdunensis* clinical isolates have demonstrated tolerance to vancomycin and related glycopeptides, although the isolates' typical susceptibility to other classes of antimicrobials eases current therapeutic concerns (Frank et al., 2008). Ongoing surveillance for acquisition of multidrug resistance characteristics by vancomycin tolerant clones of *S. lugdunensis* is important in clinical settings.

In animals, a number of case reports and a recent case-control study demonstrate a variety of infections in pets, predominantly dogs but also cats, birds, and small mammalian “pocket” pets (Briscoe et al., 2009; Beck et al., 2012; Nakamura et al., 2012; Rook et al., 2012). One of these case reports described a methicillin-resistant *S. lugdunensis* (*mecA* gene not tested) isolated from a dog that died of vegetative endocarditis (Nakamura et al., 2012). In the case-control study, *S. lugdunensis* cases were compared to *S. pseudintermedius* controls, and cases demonstrated associations with respiratory or skin infections, in-patient status, and prior use of steroids (Rook et al., 2012). *S. lugdunensis* also has been isolated from carriage sites in healthy animals (Kasprowicz et al., 2011; Beck et al., 2012). Like *S. aureus*, *S. lugdunensis* has been observed in food-producing animals (specifically sheep and goats), but the frequency of such occurrence is unknown (Deinhofer and Pernthaner, 1995; Zhang et al., 2009).

*S. lugdunensis* appears to be genetically conserved (Hellbacher et al., 2006; Frank et al., 2008; Chassain et al., 2012; Liu et al., 2012). Both *S. schleiferi* and *S. lugdunensis* have been shown to produce toxin genes, including staphylococcal enterotoxins (SEs) and toxic shock syndrome toxin (Udo et al., 1999; de Oliveira Calsolari et al., 2011). However, the prevalences for and origins of SE or TSST within these CNS species are largely unknown. The accessory gene regulator (*agr*) locus, responsible for regulation of SE and other virulence factors, is similar in *S. lugdunensis*, *S. aureus*, and veterinary pathogen *S. (pseud-)intermedius* (Frank et al., 2008). Homology between cadmium resistance genes *cadD* and *cadX* carried by *S. aureus* and *S. lugdunensis* suggests a genetic transfer event from *S. lugdunensis* plasmid pLUG10 to *S. aureus* plasmid pRW001 (Frank et al., 2008). Although the clinical importance of this particular transfer is unclear, it demonstrates the potential for horizontal gene transfer between these bacterial species.

The genome of *S. lugdunensis* has been sequenced twice, demonstrating a 78% homology with *S. aureus*, but no plasmids were identified in the two isolates, which were selected from human cases of skin infection (Tse et al., 2010; Heilbronner et al., 2011). The lack of plasmids may be due to the presence of a CRISPR region in the N920143 isolate genome, shared with *S. epidermidis* and associated with low rates of horizontal gene transfer (Heilbronner et al., 2011). However, N920143 was archived in the early 1990s and may not represent the current status of *S. lugdunensis* isolates responsible for clinical infections; for example, it contained no enterotoxin genes (Heilbronner et al., 2011). *S. lugdunensis* is distinct from other staphylococci in production of non-ribosomal protein synthetases and unusual surface proteins, although the effects of these characteristics on virulence are unknown (Heilbronner et al., 2011). Genomic comparison between *S. lugdunensis* and other sequenced genomes of *Staphylococcus* revealed presence of an *isd* iron uptake locus also linked to virulence, as well as genes responsible for producing sphingomyelinase beta-toxin (*hlb*) and haemolysin III, similar to *S. aureus* (Heilbronner et al., 2011). Studies performed prior to genomic sequencing support the ability of *S. lugdunensis* to produce a delta-hemolysin and to form biofilms (Frank et al., 2008). Genetic results suggest both new and conserved mechanisms of virulence within *S. lugdunensis* independent of its relatively low burden of antimicrobial resistance.

While CNS typically have been considered to be less pathogenic than CPS, virulence traits and demonstrated pathogenicity for *S. schleiferi* and *S. lugdunensis* deserve concern. Laboratory methods, particularly those based on phenotypic characteristics, may fail to differentiate these species from each other and from *S. aureus*, and at least one mistake in identification has been reported (Pereira et al., 2011). *S. schleiferi* and *S. lugdunensis* may resemble *S. aureus* in colony morphology on blood agar, and *S. lugdunensis* may possess similar biochemical characteristics, e.g. a positive clumping factor test (bound coagulase) that can produce erroneous speciation results by latex agglutination (Frank et al., 2008; Pereira et al., 2010). Additionally *S. schleiferi* subsp. *coagulans* is positive by tube coagulase testing, similar to *S. aureus*. Unlike *S. aureus*, both *S. schleiferi* and *S. lugdunensis* lack protein A (Vandenesch et al., 1994; Frank et al., 2008). Multiplex PCR using primers for species-specific nuclease (*nuc*) genes can differentiate *S. lugdunensis* and *S. schleiferi* from each other and from both *S. aureus* and *S. pseudintermedius* (Sasaki et al., 2010; Hirotaki et al., 2011). However, automated systems for clinical laboratory analysis may differ in their ability to identify these species correctly.

Clinical microbiologists are uniquely positioned to conduct surveillance for potentially zoonotic CNS pathogens. While high-risk samples from people, such as blood cultures, may be screened for CNS, the same attention may not be given to samples from diseased skin. Particularly in research hospital settings, laboratory testing for antimicrobial resistance patterns, including *mecA* testing and SCC*mec* typing where appropriate, is important to monitor CNS for changes in susceptibility that may impact the response to therapy. In particular, co-colonization by CNS and CPS (such as *S. aureus* and *S. pseudintermedius*) should be noted due to the potential for horizontal gene transfer. Harmonized surveillance between clinical microbiology laboratories that serve human and animal patients is essential for improved recognition of changing antimicrobial susceptibility and virulence characteristics among the CNS species that infect both people and animals.
